# Effectiveness of a Spectacle Lens with a Specific Asymmetric Myopic Peripheral Defocus: 12-Month Results in a Spanish Population

**DOI:** 10.3390/children11020177

**Published:** 2024-02-01

**Authors:** Miguel Ángel Sánchez-Tena, Jose Miguel Cleva, Cesar Villa-Collar, Marta Álvarez, Alicia Ruiz-Pomeda, Clara Martinez-Perez, Cristina Andreu-Vazquez, Eva Chamorro, Cristina Alvarez-Peregrina

**Affiliations:** 1 Department of Optometry and Vision, Faculty of Optics and Optometry, Universidad Complutense de Madrid, 28037 Madrid, Spain; masancheztena@ucm.es (M.Á.S.-T.); alicru04@ucm.es (A.R.-P.); cristina_alvarez@ucm.es (C.A.-P.); 2 ISEC LISBOA-Instituto Superior de Educação e Ciências, 1750-179 Lisbon, Portugal; clara.perez@iseclisboa.pt; 3 Clinical Research Department, Indizen Optical Technologies, 28002 Madrid, Spain; malvarez@iot.es (M.Á.); evachamorro@iot.es (E.C.); 4 Faculty of Biomedical and Health Science, Universidad Europea de Madrid, 28670 Madrid, Spain; cristina.andreu@universidadeuropea.es

**Keywords:** myopic peripheral defocus lenses, children, efficacy, myopia

## Abstract

Background: Different designs of ophthalmic lenses have been studied to control the progression of myopia in children. This study aims to evaluate the short-term efficacy of a new design of ophthalmic lens with asymmetric myopic peripheral defocus (MPDL) on myopia progression in children compared to a control group wearing a single-vision lens (SVL). Methods: Children aged 5 to 12 with myopia up to −0.50 D, astigmatism and anisometropia under 1.50 D, and corrected visual acuity over 20/20 were randomized to either the study group (MPDL) or control group (SVL). The myopia progression was evaluated by measuring axial length (AL) growth (IOL Master; Zeiss) over a period of one year. Results: Ninety-two subjects were recruited. Forty-six children were randomly assigned to the control group, and 46 to the study group. In total, 83 children completed the clinical trial, with a mean age of 10.81 [9.53–11.92] years, among which 59.04% were female. After one year of treatment, there was less AL elongation in the study group compared to the control group (0.14 ± 0.14 mm vs. 0.23 ± 0.15 mm, *p* = 0.014). Conclusions: The MPDL significantly reduced the absolute growth of AL by 39% (*p* = 0.014) and relative growth of AL by 37.3% (*p* = 0.012) after 12 months in comparison to the control group in a Spanish population.

## 1. Introduction

Myopia is one of the most common eye conditions [1,2,3] and its prevalence is increasing worldwide [4,5]. The onset of myopia in early childhood is closely related to high myopia in adulthood [5,6]. Low myopia (up to −2.75 D) and moderate myopia (−3.00 D to −5.75 D) have the potential to progress to high myopia (−6.00 D and more) with a corresponding axial length (AL) of around 26.0 mm [7] without appropriate intervention, which can have severe consequences for an individual’s ocular health. High myopia is associated with a greater risk of developing retinal detachment, myopic macular degeneration, glaucoma, and, in some cases, lead to blindness [8].

It is expected that the prevalence of high myopia will increase substantially worldwide, with its incidence expected to grow from 399 million in 2020 to 516 million by 2030 [3,9]. Consequently, pathological myopia is predicted to become the leading cause of permanent vision impairment and blindness worldwide. This growing awareness of the potential complications of high myopia has led to a global call for myopia control strategies. Therefore, it is important to detect myopia at an early stage and take measures to decrease its incidence and progression, especially among young children.

Myopia onset and progression are now understood to result from a complex interplay of visual, environmental, and genetic factors that regulate the growth of the eye in response to visual stimuli [10]. Animal studies have played a crucial role in helping us to understand myopia and develop treatment strategies [11]. Animal models have shown that emmetropization is an active process based on visual feedback and have robustly demonstrated that inducing myopic defocus inhibits eye growth, while hyperopic defocus promotes it [12]. Research with various species, including rhesus monkeys [13], marmosets [14], chicks [15], and guinea pigs [16], indicates that myopic defocus, achieved with a dual-power or multifocal lens, can inhibit or even reverse myopic eye growth. Notably, myopic defocus is considered to be the key mechanism in several contemporary myopia control strategies, including orthokeratology, multifocal soft contact lenses, and spectacle lenses.

A recent meta-analysis assessed the comparative efficacy of different optical and pharmacological interventions, showing that they may slow refractive change and reduce AL elongation [17]. Optical interventions such as ophthalmic lenses, soft contact lenses, and orthokeratology aim to alter retinal defocus by inducing myopic defocus to slow myopia progression. These optical interventions were evaluated in a meta-analysis that concluded they are effective in controlling childhood myopia progression; however, treatment effects were greater during the first 12 months of treatment and reduced over time [18].

The new designs of ophthalmic lenses recently developed to control myopia progression can be divided into two groups: peripheral myopic defocus and diffusion optics technology. Both designs are based on the two mechanisms studied for myopia progression: peripheral hyperopic defocus and abnormal cone contrast signaling in the retina [19]. Within the first category, we can include progressive peripheral defocus ophthalmic lenses [20,21,22,23] and defocus incorporated multiple segments (DIMS) [24], highly aspherical lenslets target (HALT) [25,26], and cylindrical annular refractive element (CARE) [27] spectacle lenses, while the second category includes diffusion optics technology (DOT) spectacle lenses [28].

This study presents a new ophthalmic lens design based on a specific asymmetric myopic peripheral defocus (MPDL), with an ovoidal blur-free central area surrounded by a progressive power distribution producing asymmetrical myopic defocus.

The study aims to evaluate if the proposed design can slow myopia progression in schoolchildren compared to a control group corrected with a single-vision lens (SVL).

## 2. Materials and Methods

A prospective, double-blind, randomized clinical trial (RCT) was carried out to evaluate the efficacy of a new ophthalmic lens design developed by IOT for myopia control at the Novovision Clinic in Madrid (Madrid, Spain).

The study included children aged between 5 to 12 years old who met certain criteria: cycloplegic spherical equivalent (SE), defined as sphere + ½ cylinder, had to be lower than −0.50 D; astigmatism had to be less than 1.50 D; anisometropia had to be less than 1.50 D, and visual acuity had to be equal to or greater than 20/20. Children who had previously received any treatment for myopia control, had any ocular pathology, were using drugs that could affect pupil size, or had any systemic disease that could affect vision were excluded from the study.

The study recruited 100 myopic children who were randomly assigned to either the treatment or control group. The treatment group wore the MPDL, while the control group wore a standard spherical SVL.

**Figure 1:**
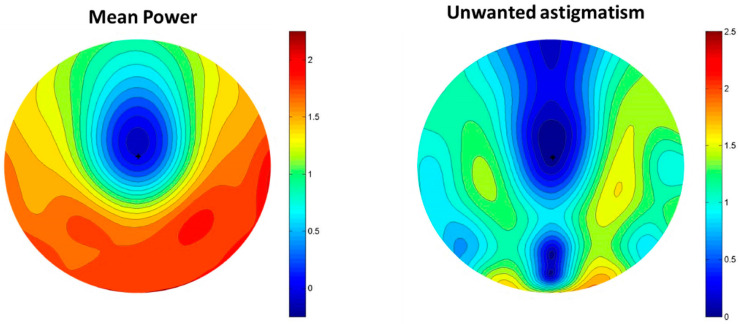
Spherical equivalent and cylinder power distribution maps for a plano prescription of the new design of ophthalmic lens for myopia control.

The MPDL comprised an ovoidal blur-free central area with a horizontal size of 7 mm and a peripheral treatment area consisting of an asymmetrical horizontal myopic defocus with an addition value of +1.50 D at 25 mm nasally and +1.80 D at 25 mm temporally. Additionally, the lens also featured a myopic defocus inferiorly with a value of +2.00 D. The spherical equivalent and cylinder power distribution maps for a plano prescription are shown in Figure 1.

All lenses were manufactured by free-form technology in index 1.6 with anti-reflective coating considering the children’s prescription, monocular pupillary distances, and pupil heights. FreeFormDesigner software version 10.5 (IOT, Madrid, Spain) was used for all lens calculations.

Lenses were fitted to spectacle frames that ranged in horizontal size from 41 mm to 55 mm and in vertical size from 30 mm to 48 mm. As would be expected and advised by the optician, younger children generally selected smaller frames than older children.

The procedures followed in the RCT and the instruments used were:

- Sociodemographic questionnaire: Participants’ parents completed a questionnaire that included information regarding age (date of birth), gender, ocular and medical health records, ocular surgeries, history of parents’ myopia, age of myopia onset, history of myopia progression, and previous eye treatments.
- Objective refraction: Performed using an autorefractometer after cycloplegia using 3 drops of cyclopentolate.
- Biometry: AL was measured with the optical biometer IOL Master^®^ (Carl Zeiss Meditec, Jena, Germany)
- Binocular vision: Distance and near vision phoria were measured using Von Graefe’s technique, the amplitude of accommodation was measured using the Sheard method, and the accommodative lag was measured using MEM retinoscopy.
- Wearability questionnaires: All of the children, with parental assistance, completed questionnaires to assess their satisfaction with the spectacles, record the time of use, and document any issues associated with the spectacles or any adverse effects.

During the first visit, the optometrist provided information about the study to the parents of the children, who then signed a written consent form after any doubts were addressed. The optometrist also checked that the children met the inclusion criteria. Baseline measurements were also taken. All children underwent an ophthalmology exam to evaluate their ocular health, and their cycloplegic autorefraction, binocular vision, and AL were recorded in the case report form. Additionally, parents completed a sociodemographic questionnaire, and the children selected frames for their spectacles.

The second visit was scheduled for the collection of the spectacles, which the children were required to wear throughout the day. The optometrist checked the fitting of the spectacles and gave each child a wearability questionnaire to complete and return at the next visit. Children whose refraction changed by more than 0.50 D during the study or who suffered an adverse incident, such as broken frames or scratched lenses, were provided with a new pair.

Wearability questionnaires were collected at 6 and 12 months following the second visit, and the children also underwent a cycloplegic autorefraction, binocular vision, and AL assessment, which was recorded in the case report form.

Table 1 shows a summary of the data collected at each visit.

The RCT was approved by the Institutional Ethics Committee of Hospital Clínico San Carlos (Madrid, Spain) and adhered to the tenets of the Declaration of Helsinki. The trial was registered on ClinicalTrials.gov (accessed on 25 January 2024) with ID NCT05250206.

A comparative statistical analysis of absolute (mm) and relative (%) AL growth between the MPDL and SVL groups was performed using STATA^®^ software v.14. Absolute AL growth was calculated as the median relative difference after 6 and 12 months of treatment: (AL at 6 m − baseline AL)/baseline AL × 100 and (AL at 12 m − baseline AL)/baseline AL × 100. The significance level was set as *p*-value < 0.05. The efficacy of the MPDL was evaluated by comparing the means of relative AL growth in children treated with MPDL to that of children treated with SVL.

## 3. Results

**Figure 2:**
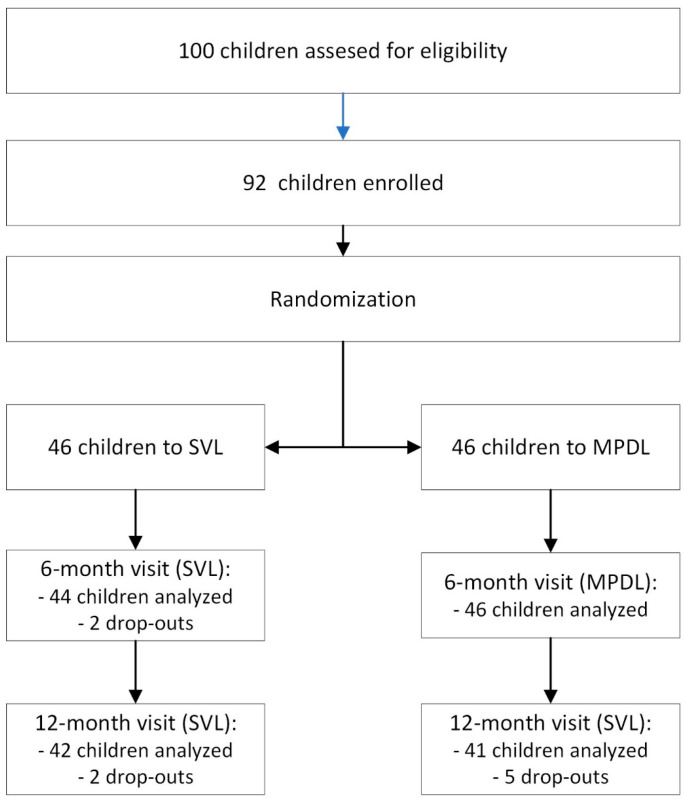
Randomized clinical trial flow chart with participant randomization, treatment group assignment, and follow-up visits. SVL: Single-vision lens; MPDL: Asymmetric myopic peripheral defocus lens.

Out of 100 children who were assessed for eligibility, 92 were enrolled and randomized into two groups. The control group consisted of 46 children using SVL, and the study group included 46 children wearing MPDL. The flowchart in Figure 2 shows the enrollment and follow-up visits, indicating that 9 of the participants did not complete the study (4 in the SVL group and 5 in the MPDL group), resulting in a dropout rate of 9.8%. Finally, the 83 children analyzed were divided into 42 (50.6%) in the control group with SVL and 41 (49.4%) in the study group with MPDL.

Table 2 presents the baseline parameters of the entire sample and each study group. The age of the children who participated in the study ranged from 6.4 to 13.7 years, with 59.0% being girls. The initial axial length in the study group was 24.17 ± 0.91 mm, while it was 23.98 ± 0.82 mm in the control group. As for the initial spherical equivalent, it was −2.63 ± 1.28 D in the MPDL group and −2.01 ± 1.03 D in the SVL group.

**Figure 3:**
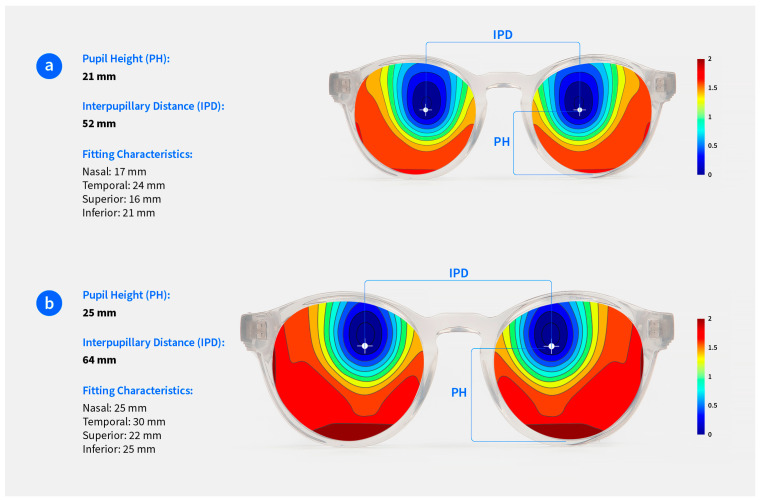
Fitting characteristics of 2 participants of the study with extremely different face physiognomies, indicating distances from the pupil to the nasal, temporal, superior, and inferior sides of the edge of the frame. (**a**) Seven-year-old participant wearing a frame with horizontal size of 41 mm, vertical size of 37 mm, and bridge of 18 mm. (**b**) Eleven-year-old participant wearing a frame with horizontal size of 55 mm, vertical size of 47 mm, and bridge of 14 mm.

The fitting characteristics of the children varied as a result of different face physiognomies, leading to a range of distances from the pupil to the nasal side of the frame of 20 ± 2 mm (from 16 to 24 mm), to the temporal side of 30 ± 3 mm (from 24 to 35 mm), to the superior side of 14 ± 4 mm (from 8 to 23 mm), and to the inferior side of 26 ± 3 mm (from 20 to 32 mm). Figure 3 shows an illustrative example of the fitting characteristics of 2 participants of the study with extremely different face physiognomies, indicating the distances from the pupil to the nasal, temporal, superior, and inferior sides of the edge of the frame. In example A, the maximum positive defocus was 1.78 D, and in example B, the maximum positive defocus was 1.98 D.

Based on subjective prescription changes over or equal to 0.25 D during the 12-month duration of the study, the optical prescription was updated in 18 children from the SVL group and 15 children from the MDPL group. Additionally, 1 child in the SVL group and 0 children in the MDPL group required more than one change of lenses. The mean change in prescription was −0.36 ± 0.43 D in the SVL group and −0.25 ± 0.18 D in the MDPL group.

After 6 months of treatment, the SE increased by a mean of 0.07 ± 0.22 diopters in the group of children who wore MPDL, while it increased by −0.09 ± 0.27 diopters in the group of children who wore SVL. The difference in the increase between the two groups was found to be statistically significant, with a *p*-value of 0.0034.

One year after treatment, the SE increased by −0.00 ± 0.31 diopters in the MPDL group and −0.25 ± 0.32 diopters in the SVL group. The difference in the increase between the two groups was found to be statistically significant, with a *p*-value of 0.0005. These changes represented a relative growth of 1.0 ± 16.1% in the MPDL group versus 16.4 ± 19.1% in the SVL group (*p* = 0.0002).

Table 3 shows the AL values for each group in all visits, as well as the relative changes.

**Figure 4:**
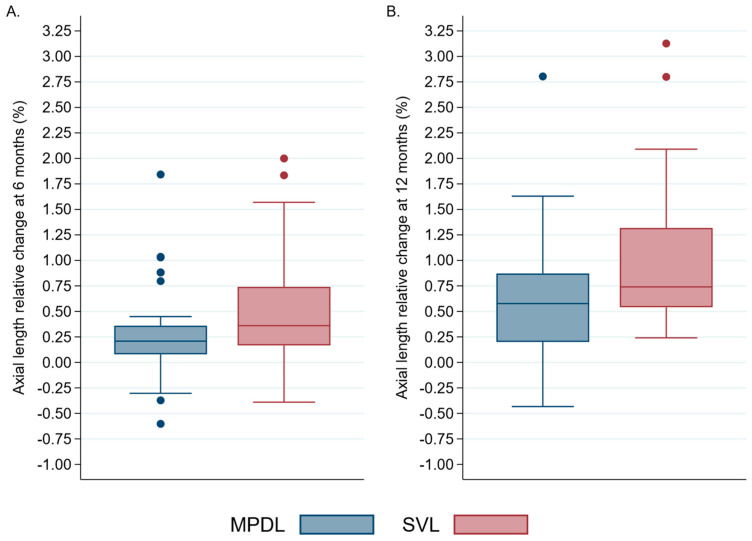
(**A**) Axial length relative change at 6 months (%), and (**B**) axial length relative change at 12 months (%). SVL: Single-vision lens; MPDL: Asymmetric myopic peripheral defocus lens.

After 6 months, the AL of the eye increased by 0.06 ± 0.09 mm in the group that received MPDL, while it increased by 0.12 ± 0.11 mm in the group that received SVL (*p* = 0.018). This resulted in a relative growth rate of 0.20% [0.07–0.35] in the MPDL group compared to 0.36% [0.16–0.74] in the SVL group (*p* = 0.02). After 1 year, the AL increased by 0.14 ± 0.14 mm in the MPDL study group and by 0.23 ± 0.15 mm in the SVL control group (*p* = 0.014), representing a relative growth of 0.57% [0.20–0.87] in the MPDL group versus 0.74% [0.54–1.31] in the SVL group (*p* = 0.01). Figure 4A,B visually display these relative changes.

The efficacy of the MPDL was evaluated by comparing the means of relative AL growth in children treated with MPDL to that of children treated with SVL. During the first 6 months of treatment, children in the MPDL group showed a mean value of relative increase in AL of 0.27 ± 0.42%, while children in the SVL group showed a mean value of relative increase of 0.51 ± 0.53%. The MPDL proved to significantly reduce the relative growth of AL by 46.9% (*p* = 0.026).

After 12 months, the mean value of relative increase in AL was 0.98 ± 0.65% for children in the SVL group and 0.61 ± 0.64% for children in the MDPL group. The MPDL significantly reduced the relative growth of AL by 37.3% (*p* = 0.012).

Regarding the factors associated with relative AL growth, univariate linear regression analyses indicated that factors such as sex, presence of one or two parents with myopia, time spent outdoors, hours spent doing homework or in front of the computer, daily hours of sleep, and initial spherical equivalent did not have significant impacts on the relative growth of AL over 6 and 12 months of treatment. However, it was observed that the age of the child at the beginning of the study influenced the relative growth of AL in both groups (6-month regression coefficient: −0.066, 95% CI: −0.124 to −0.007; 12-month regression coefficient: −0.134, 95% CI: −0.210 to −0.058), with the relative growth being lower in children with MPDL compared to children with SVL (regression coefficient at 6 months: −0.238, IC 95%: −0.447 a −0.029; regression coefficient at 12 months: −0.364, IC 95%: −0.646 a −0.083).

**Figure 5:**
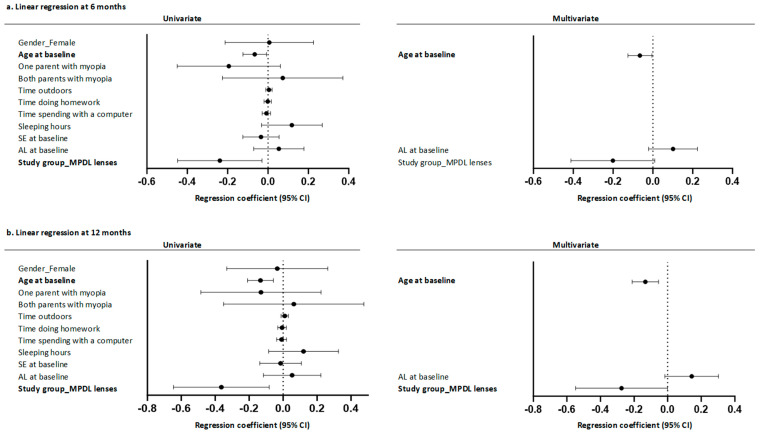
Factors associated with the relative growth of axial length at (**a**) 6 months and (**b**) 12 months following the start of the study.

Multivariate analysis confirmed the effect of age, showing that, for each additional year of the child’s age at baseline, relative growth was lower at both 6 months (regression coefficient: −0.065, 95% CI: −0.125 to −0.004) and 12 months of treatment (regression coefficient: −0.133, 95% CI: −0.212 to −0.054). In addition, multivariate regression analysis revealed that regardless of the child’s age and AL at the beginning of treatment, the relative growth of AL in children in the MPDL group tended to be lower after 6 months (regression coefficient: −0.238, 95% CI: −0.447 to −0.029) and was significantly lower at 12 months (regression coefficient: −0.275, 95% CI −0.548 to −0.001) compared to that in children in the SVL group (Figure 5). 

## 4. Discussion

The use of spectacle lenses for managing the progression of myopia has been well studied in recent years, demonstrating different levels of efficacy between different lens designs.

Sankaridurg et al. [20] showed no significant differences in the rate of progression of myopia in Chinese children between the control group that used SVL and the group that used one of three types of lens based on reducing peripheral hyperopic defocus, using central apertures of 20 mm, 14 mm, and 10 mm in diameter combined with a positive additional peripheral power of 1 D, 2 D, or 1.9 D, respectively. Later, Kanda et al. [22] tested MyoVision lenses, which have an asymmetric design with a central aperture of 20 mm in diameter and a positive additional peripheral power of 1.9 D. The study performed with Japanese children did not show significant differences in the progression of myopia or ocular elongation between the control group (SVL) and the group wearing MyoVision lenses.

Although these lenses seemed to not have enough efficacy to reduce myopia progression, there are other studies with different designs of ophthalmic lenses that have shown effectiveness in controlling AL progression in juvenile-onset myopes, such as peripheral progressive defocus lenses (perifocal and Shamir Myopia Control lenses) [21,23], lenslets (DIMS and HALT) [24,25], rings (CARE) [27], or diffusion optics (DOT) [28], not only in Asian populations [24,25,27] but also in Caucasian populations [21,29].

Analyzing the results of the above-mentioned studies in more detail, perifocal lenses were studied in a Russian population, showing significant differences against SV lenses. These peripheral progressive defocus lenses consisted of a central area for full prescription correction of 10 mm in diameter surrounded by asymmetric horizontal progressive defocus reaching a maximum addition of 2.50 D at 25 mm of the temporal side and +2.00 D at 25 mm of the nasal side [21].

Shamir Myopia Control lenses were studied in children from Israel, showing significant differences in the progression of AL during 1 year of follow-up compared to an SVL group. This design has a similar power distribution to the one used in our study, with a central region for full prescription correction with a horizontal width of 10 mm and a positive power that gradually increases up to 1–3 D at different peripheral areas of the lens [23].

DIMS technology has been thoroughly researched, with data available for up to 6 years in Chinese children [30]. The DIMS spectacle lens has a central aperture of 9 mm in diameter for full prescription correction surrounded by a treatment area of 33 mm in diameter with multiple lenslets of 3.5 D positive power [31]. The data from 6 years of follow-up on DIMS lenses demonstrated that there was no rebound effect when the treatment stopped, with myopia control maintained throughout the entire study duration [30]. DIMS lenses have also demonstrated similar efficacy in European children and adolescents treated with a combined method of DIMS and atropine [29].

HALT lenses include a central area for full prescription correction with a central aperture of 10 mm in diameter surrounded by a treatment area with 11 concentric rings formed by aspherical lenslets (diameter of 1.1 mm) with positive power between 3.50 D for lenslets of the peripheral ring and 6.00 D for central rings. HALT lenses have also undergone thorough examination in Chinese children, with data available for up to 4 years [32]. Another study on HALT spectacle lenses in Vietnamese children concluded their effectiveness in slowing myopia progression, with no indication of myopia rebound upon discontinuation [33].

CARE technology consists of a central optical area for myopia correction and a treatment area formed by concentric rings. The design for children under 10 has a 7 mm diameter central zone and a mean surface power of +4 D, and the design for children older than 10 has a 9 mm diameter central zone and a mean surface power of +3.80 D [27]. CARE lenses significantly reduced AL elongation in Chinese children over 1 year compared with SVL [27].

Finally, DOT technology consists of a 5 mm diameter central zone surrounded by translucent microscopic diffusers (diameter of 0.14 mm) to scatter light, with the intent of reducing contrast and therefore reducing the relative activity difference between L and M cones. Two investigational DOT lenses with two different diffusion densities (test 1 and test 2) have been studied, showing effectiveness in controlling myopia progression and axial elongation in North American children compared with SVL [28].

The lens used in this study, MPDL, was developed based on hyperopic defocus theory, with an oval central zone of 7 mm in horizontal width providing full prescription correction surrounded by an asymmetric peripheral defocus treatment area, reaching an addition of +1.50 at 25 mm nasally, +1.80 D at 25 mm temporally, and +2.00 D at the bottom of the lens. After 1 year of treatment, the MPDL study group showed an average eye growth of 0.14 mm, and the control group showed 0.23 mm (*p* = 0.014), corresponding to an absolute efficacy of 39%. The progressions in the control and treatment groups were less than the average obtained with Asian populations using DIMS, HALT, and CARE lenses (change in AL from 0.30 to 0.36 mm). This could be due to the differences between the ethnicities and ages of the populations studied, with the average age of the children in this study being slightly older. It has been demonstrated that the progression of myopia depends on age and race, with AL elongation decreasing as age increases and greater in Asian children compared to non-Asian children [34]. However, studies with Caucasian populations have shown similar rates of AL progression in children not treated with methods to control the progression of myopia, with progression of 0.24 mm in Spanish children [35] and 0.34 mm in 9-year-old European myopes [36].

Our results showed an absolute AL change between the MPDL and SVL groups of 0.09 mm over 1 year. With the same follow-up duration, several studies using peripheral progressive defocus lenses in Chinese [20] and Japanese [22] populations have not shown statistically significant results in the progression of myopia compared to the use of SVL. However, the results of Tarutta et al. [21] with perifocal lenses in Russian children and Yuval et al. [23] with Shamir Myopia Control lenses in Israeli children showed similar results, with an AL change between the study lens group and the SVL group of 0.09 mm and 0.11 mm, respectively. In comparison with other myopia management spectacle lenses, our results in a Spanish population showed, in general, lower AL change between the MPDL and SVL groups than in studies performed in other regions with other lenses. Lam et al. [24] showed a difference in AL progression between the DIMS group and SVL group of 0.21 mm in Chinese children. Bao et al. showed a difference in AL progression between the SVL control group and HALT group of 0.23 mm [25] in Chinese children. Liu et al. showed that treatment with CARE lenses significantly reduced the rate of AL elongation, with a difference of 0.10 mm between the CARE and SVL groups [27] in Chinese children. And Rappon et al., in the CYPRESS study with North American children, demonstrated that DOT test lenses significantly reduced the progression of AL by averages of 0.15 mm and 0.11 mm for the test 1 and 2 lenses, respectively, compared to SVL [28].

The efficacy results of myopia control treatments have historically been presented as percentage of efficacy, involving a simple comparison between the increase occurring in the control group versus the study group. This percentage of reduction in progression as an index to describe treatment effect has been considered misleading. Given the influence of certain parameters, such as children’s age and treatment duration, recent studies recommend showing the efficacy of a lens based on the relative growth of AL [37]. In this sense, the MPDL showed a 37.3% decrease in the relative growth of AL. The difference in efficacy between the different ophthalmic lens studies could be due to various reasons, such as differences in the designs of the lenses used, differences in the characteristics of the study population (age, ethnicity, etc.), or differences in the methodology used. In this sense, for improved accuracy and relevance, this study has used relative growth to calculate efficacy, therefore the initial values of AL have been considered.

Regarding the lens design, most of the lenses previously described have a central area providing full prescription correction (of greater or smaller diameter depending on the design) and a peripheral area with positive addition that aims to create a myopic blur in the peripheral retina. All of the designs of these spectacle lenses (MPDL, DIMS, HALT, SMC, perifocal, and CARE lenses) align with the theory of creating simultaneous myopic retinal defocus during both distance and near viewing with an optical central zone between 7 and 10 mm in diameter surrounded by an addition range from 1.50 D to 3.80 D. Smaller central zone diameters could cause vision problems and discomfort when wearing the lenses. Another aspect to consider in lens design is the myopic defocus, the magnitude and distribution of which could directly impact the effectiveness of the lens. Previous studies have suggested that creating different retinal peripheral defocuses could play an important role in controlling myopia [38,39], and it has been shown that using high add power multifocal contact lenses resulted in a considerable decrease in the rate of myopia progression when compared to medium add power [40].

On the other hand, the existing research does not include any trials of these new designs of ophthalmic lenses in the Spanish population. There are three studies with Caucasian populations, two of them evaluated the efficacy of DIMS in slowing the progression of myopia, the first in German children combined with atropine [29] and the second in English children [41], and the other showed the effect of wearing perifocal glasses in Russian children [21]. In this sense, our study is the first to show efficacy data for a spectacle lens for myopia management in Spanish subjects.

Regarding the limitations of this study, one of them is the absence of stratification in the randomization process, which led to differences in both initial age and initial refraction. The second one is the age range of the sample; future research would ideally involve analyzing the results by age group. The follow-up time should also be extended, as it has been observed in many studies [18] that the effectiveness during the first year of treatment is greater than that in the second and following years of follow-up. Future research should focus on increasing the follow-up period to show the long-term efficacy of the MPDL and any possible rebound effects. This extension has already been approved by the ethics committee. Finally, it would be also interesting for future analysis to examine the influence of the frame and fitting characteristics on the efficacy of the lens, as well as the influence of the convergence in the adaptation and efficacy of the MDPL.

In summary, today myopia is not only treated as a refractive error but also as an ocular disorder that requires effective intervention. To prevent severe myopia and its associated complications, various optical treatments have been explored to slow down the progression of myopia, showing clinically meaningful reductions in myopia progression [42]. Myopia management spectacles are a popular choice because they can be easily fitted even for younger children. Moreover, spectacles have minimal to no side effects in comparison to alternative optical treatments like contact lenses or pharmaceutical therapies such as atropine. The optimal intervention will be clinically selected based on effectiveness and depending on the geographical location [11,31].

Related to spectacle lenses to control the progression of myopia in children, the current study provides the first randomized, double-blind study of a 1-year clinical trial carried out in a European population, with Spanish children wearing an asymmetric myopic defocus ophthalmic lens design, showing that the study group experienced significantly less ocular elongation compared to the SVL group.

## 5. Conclusions

The MPDL is effective in myopia control in a Spanish population, showing reductions in absolute growth of AL by 50% and 39% after 6 and 12 months of treatment, respectively, and in relative growth of AL by 46.9% and 37.3% after 6 and 12 months of treatment, respectively.

## Figures and Tables

**Table 1:** Schedule of assessments and examination items.

Visit		V0	V1	V2	V3
Consent form signed		X			
Basic Information	Demographics	X			
	History	X			
Refraction	Subjective Refraction	X		X	X
	Cycloplegic autorefraction	X		X	X
Visual acuity	Uncorrected visual acuity	X		X	X
	Best corrected visual acuity	X		X	X
Eye examination	Keratometry	X			
	Slit lamp examination	X			
	Fundus examination	X			
	Intraocular pressure measurement	X			
	Axial length	X		X	X
Binocular vision	LAG	X		X	X
	Accommodation Amplitude	X		X	X
	Phoria (distance and near)	X		X	X
Spectacle fitting	Frame position and lens condition		X		
Questionnaire	Lifestyle	X			
	Wearability			X	X

**Table 2:** Baseline characteristic of the sample.

Baseline Characteristics *	Total (n = 83)	MPDL (n = 41)	SVL (n = 42)	*p*-Value **
Age (Median [Q1, Q3])	10.81 [9.53–11.92]	11.7 [9.97–11.98]	10.49 [8.76–11.32]	0.022
Gender (n, %)				0.212
Boys	41.0 (34)	34.1 (14)	47.6 (20)	
Girls	59.0 (34)	65.9 (27)	52.4 (22)	
Initial cycloplegic refraction (Mean ± SD)	−2.32 ± 1.20	−2.63 ± 1.28	−2.01 ± 1.03	0.018
Initial axial length (Mean ± SD)	24.07 ± 0.86	24.17 ± 0.91	23.98 ± 0.82	0.329
Myopic parents (%, n)				0.657
0	40.3 (31)	36.8 (14)	43.6 (17)	
1	37.7 (29)	36.8 (14)	38.5 (17)	
2	22.1 (17)	26.3 (10)	18.0 (7)	
Hours/week outdoors (Median [Q1, Q3])	6.5 [2.5–10]	6 [3–10]	7 [2–10.50]	0.604
Hours/week doing homework (Median [Q1, Q3])	6 [2.5–10]	6 [2.5–12]	5.5 [3–10]	0.697
Computer Hours/Week (Median [Q1, Q3])	3 [0.5–8]	4 [1–7]	2.25 [0–8]	0.652
Sleeping hours (Mean ± SD)	9.58 ± 0.71	9.52 ± 0.59	9.64 ± 0.82	0.461

* Weekday hours during the school term. ** *p*-value of the comparison between MPDL and SVL groups (Chi-square test for qualitative variables, or Student’s *t*-test for independent samples or Mann–Whitney U test, according to normality, for quantitative variables.

**Table 3:** Axial length by group in all visits.

	MPDL (n = 41)	SVL (n = 42)	*p*-Value
Initial axial length (Mean ± SD)	24.17 ± 0.91	23.98 ± 0.82	0.329
6-month visit axial length (Mean ± SD)	24.23 ± 0.93	24.10 ± 0.84	0.507
12-month visit axial length (Mean ± SD)	24.32 ± 0.95	24.22 ± 0.85	0.610
**Axial Length Change at 6 Months**			
Absolute axial length change (mm)	0.06 ± 0.09	0.12 ± 0.11	0.018
Relative change in axial length (Mean)	0.27 ± 0.42	0.51 ± 0.53	0.026
Relative change in axial length (Median [Q1, Q3]) (AL at 6 months—AL at baseline/AL at baseline) × 100 (%)	0.20 [0.07–0.35]	0.36 [0.16–0.74]	0.020
**Change in Axial Length at 12 Months**			
Absolute axial length change (mm)	0.14 ± 0.14	0.23 ± 0.15	0.014
Relative change in axial length (Mean)	0.61 ± 0.64	0.98 ± 0.65	0.012
Relative change in axial length (Median [Q1, Q3]) (AL per year—AL at baseline/AL at baseline) × 100 (%)	0.57 [0.20–0.87]	0.74 [0.54–1.31]	0.010

SVL: Single-vision lens; MPDL: asymmetric myopic peripheral defocus lens.

## Data Availability

The data presented in this study are available on request from the corresponding author. The data are not publicly available due to to specific ethical and privacy considerations.
