# Correction: Sánchez-Tena et al. Effectiveness of a Spectacle Lens with a Specific Asymmetric Myopic Peripheral Defocus: 12-Month Results in a Spanish Population. *Children* 2024, *11*, 177

**DOI:** 10.3390/children12030311

**Published:** 2025-02-28

**Authors:** Miguel Ángel Sánchez-Tena, Jose Miguel Cleva, Cesar Villa-Collar, Marta Álvarez, Alicia Ruiz-Pomeda, Clara Martinez-Perez, Cristina Andreu-Vazquez, Eva Chamorro, Cristina Alvarez-Peregrina

**Affiliations:** 1 Department of Optometry and Vision, Faculty of Optics and Optometry, Universidad Complutense de Madrid, 28037 Madrid, Spain; masancheztena@ucm.es (M.Á.S.-T.); alicru04@ucm.es (A.R.-P.); cristina_alvarez@ucm.es (C.A.-P.); 2 ISEC LISBOA-Instituto Superior de Educação e Ciências, 1750-179 Lisbon, Portugal; clara.perez@iseclisboa.pt; 3 Clinical Research Department, Indizen Optical Technologies, 28002 Madrid, Spain; malvarez@iot.es (M.Á.); evachamorro@iot.es (E.C.); 4 Faculty of Biomedical and Health Science, Universidad Europea de Madrid, 28670 Madrid, Spain; cristina.andreu@universidadeuropea.es

## Text Correction

There was an error in the original publication for the abstract [1]. In the abstract, the sentence was written as “After one year of treatment, there was less AL elongation in the study group compared to the control group (0.16 ± 0.16 mm vs. 0.24 ± 0.16 mm, *p* = 0.034)”, but the actual data for the manuscript should be “After one year, the AL increased by 0.14 ± 0.14 mm in the MPDL study group and by 0.23 ± 0.15 mm in the SVL control group (*p* = 0.014)”. The authors state that the scientific conclusions are unaffected. This correction was approved by the Academic Editor. The original publication has also been updated.

The corrected abstract appears below:

**Abstract:** Background: Different designs of ophthalmic lenses have been studied to control the progression of myopia in children. This study aims to evaluate the short-term efficacy of a new design of ophthalmic lens with asymmetric myopic peripheral defocus (MPDL) on myopia progression in children compared to a control group wearing a single-vision lens (SVL). Methods: Children aged 5 to 12 with myopia up to −0.50 D, astigmatism and anisometropia under 1.50 D, and corrected visual acuity over 20/20 were randomized to either the study group (MPDL) or control group (SVL). The myopia progression was evaluated by measuring axial length (AL) growth (IOL Master; Zeiss) over a period of one year. Results: Ninety-two subjects were recruited. Forty-six children were randomly assigned to the control group, and 46 to the study group. In total, 83 children completed the clinical trial, with a mean age of 10.81 [9.53–11.92] years, among which 59.04% were female. After one year of treatment, there was less AL elongation in the study group compared to the control group (0.14 ± 0.14 mm vs. 0.23 ± 0.15 mm, *p* = 0.014). Conclusions: The MPDL significantly reduced the absolute growth of AL by 39% (*p* = 0.014) and relative growth of AL by 37.3% (*p* = 0.012) after 12 months in comparison to the control group in a Spanish population.
